# A Novel Harmony Search Algorithm Based on Teaching-Learning Strategies for 0-1 Knapsack Problems

**DOI:** 10.1155/2014/637412

**Published:** 2014-01-08

**Authors:** Shouheng Tuo, Longquan Yong, Fang'an Deng

**Affiliations:** School of Mathematics and Computer Science, Shaanxi University of Technology, Hanzhong 723001, China

## Abstract

To enhance the performance of harmony search (HS) algorithm on solving the discrete optimization problems, this paper proposes a novel harmony search algorithm based on teaching-learning (HSTL) strategies to solve 0-1 knapsack problems. In the HSTL algorithm, firstly, a method is presented to adjust dimension dynamically for selected harmony vector in optimization procedure. In addition, four strategies (harmony memory consideration, teaching-learning strategy, local pitch adjusting, and random mutation) are employed to improve the performance of HS algorithm. Another improvement in HSTL method is that the dynamic strategies are adopted to change the parameters, which maintains the proper balance effectively between global exploration power and local exploitation power. Finally, simulation experiments with 13 knapsack problems show that the HSTL algorithm can be an efficient alternative for solving 0-1 knapsack problems.

## 1. Introduction

Harmony search (HS) [1, 2] is a new population-based metaheuristic optimization algorithm. It has received much attention regarding its application potential as continuous and discrete optimal problem. Inspired by the process of the musicians' improvisation of the harmony, the HS algorithm improvises its instruments' pitches searching for a perfect state of harmony. The effort to find a new harmony in music is analogous to finding a better solution in an optimization process. HS has been applied to optimization problems in different areas [3–11]. The HS algorithm has powerful exploration ability in a reasonable time but is not good at performing a local search. In order to improve the performance of the harmony search method, several variants of HS have been proposed [12–20]. These variants have some improvement on continuous optimization problems. However, their effectiveness in dealing with discrete problems is still unsatisfactory.

The knapsack problem is one of the classical combinatorial optimization problems. It derives its name from the problem faced by someone who is constrained by a fixed-size knapsack and must fill it with the most valuable items. The knapsack problem often applies to resource allocation where there are financial constraints and is studied in fields such as combinatorics, computer science, complexity theory, cryptography, and applied mathematics.

The 0-1 knapsack problem is as follows. Given a set of *D* items and a knapsack, with

(1) pj=profit  of  item  j,wj=weight  of  item  j,cj=volume  of  item  j,W=weight  capacity  of  knapsack,V=volume  capacity  of  knapsack,

select some items so that the total profit of the selected items is maximum, and the total weight and the total volumes of selected items are not more than the weight capacity and the volume capacity of the knapsack. Formally,

(2) $$\begin{array}{c} \text{maximize}\;z=\sum_{j=1}^{D}p_{j}x_{j}, \end{array}$$

subject to

(3) ∑j=1Dwjxj≤W,∑j=1Dcjxj≤V,

where

(4) $$\begin{array}{c} x_{j}=\{\begin{array}{cc} 1, & \text{if}\;\text{item}\;j\;\;\text{is}\;\text{assigned}\;\text{to}\;\text{knapsack}\\ 0, & \text{otherwise}. \end{array} \end{array}$$

Many methods have been employed to solve 0-1 knapsack problems. Zou et al. proposed a novel global harmony search algorithm (NGHS) for 0-1 knapsack problems [21]. Y. Liu and C. Liu presented an evolutionary algorithm to solve 0-1 knapsack problems [22]. Shi used an improved ant colony algorithm to solve 0-1 knapsack problems [23]. Lin solved the knapsack problems with imprecise weight coefficients by using genetic algorithm [24]. Boyer et al. solved knapsack problems on GPU [25]. Hill et al. proposed a heuristic method for 0-1 multidimensional knapsack problems [26]. Gherboudj et al. propose a discrete binary cuckoo search (BCS) algorithm in order to deal with binary optimisation problems [27]. A novel quantum inspired cuckoo search for knapsack problems is present in the literature [28].

In recent years, more and more discrete optimization problems are solved by HS method. To some extent, this is due to the memory consideration rule that is appropriate to be employed to resolve the discrete optimization problems. However, for a high-dimensional discrete optimization problem, classical HS algorithm can be easy to cause premature convergence and stagnation behavior. Therefore, we present a dynamic parameters-adjustment mechanism for solving the high-dimensional 0-1 knapsack problems. To enhance the performance of dealing with discrete problems by HS method, this paper proposed an improved HS algorithm based on teaching-learning (HSTL) strategies.

The rest of the paper is organized as follows.  Section 2 introduces the classical HS algorithm and three state-of-the-art variants of HS. The teaching-learning-based optimization (TLBO) algorithm and the proposed approach (HSTL) are introduced in Section 3.  Section 4 presents related constraint-handling technique and integer processing method. Experimental results are reported in Section 5. Finally, Section 6 concludes this paper.

## 2. HS Algorithm and Other Variants

In this section, we introduce the classical HS algorithm and three state-of-the-art variants of HS algorithms: NGHS algorithm [17], intelligent tuned harmony search algorithm (ITHS) [18], and exploratory power of harmony search algorithm (EHS) [19].

### 2.1. Classical Harmony Search Algorithm (HS)

Classical harmony search (HS) is derivative-free meta-heuristic algorithm. It mimics the improvisation process of music players and uses three rules (memory consideration, pitch adjustments, and randomization) to optimize the harmony memories. The steps in the procedure of classical harmony search algorithm are as follows.

**Step 1 (initialize the harmony memory)**

The harmony memory (HM) consists of HMS harmony. Each harmony is generated from a uniform distribution in the feasible space, as

(5)   xij=xiL+rand()·(xiU−xiL),i=1,2,…,D;  j=1,2,…,HMS,

where rand() is a uniform distribution random number between 0 and 1.

Consider the following:

(6) $$\begin{array}{c} \text{HM}=\left[\begin{array}{c} X^{1}\\ X^{2}\\ \vdots \\ X^{\text{HMS}} \end{array}\right]=\left[\begin{array}{cccc} x_{1}^{1} & x_{2}^{1} & \cdots & x_{D}^{1}\\ x_{1}^{2} & x_{2}^{2} & \cdots & x_{D}^{2}\\ \vdots & \vdots & \vdots & \vdots \\ x_{1}^{\text{HMS}} & x_{2}^{\text{HMS}} & \cdots & x_{D}^{\text{HMS}} \end{array}\right]. \end{array}$$

**Step 2 (improvise a new harmony via three rules)**

Improvise a new harmony *X* ^new^ via three rules: memory consideration, pitch adjustment, and random generation.

*(a) Memory Consideration*. Decision variable value of the new harmony will be generated by choosing from the harmony memory with probability HMCR. 

*(b) Pitch Adjustment. *Get a component randomly from an adjacent value of one decision variable of a harmony vector with probability PAR. 

*(c) Random Generation. *Generate a component randomly in the feasible region with probability 1-HMCR.

The improvisation procedure of a new harmony works as Algorithm 1.

A new potential variation (or an offspring) is generated in Step 2, which is equivalent to mutation and crossover operator in standard Evolution Algorithms (EAs).

**Step 3 (update the worst harmony)**

Consider the following:

(7) If  Xnew  is  better  than  XworstXnew=Xworst,Endif,

where *X* ^worst^ = (*x*_1_^worst^, *x*_2_^worst^,…, *x*_*D*_^worst^) denotes the worst harmony in HM.

**Step 4 (check stopping criterion)**

If the stopping criterion (maximum function evaluation times: MaxFEs) is satisfied, computation is terminated. Otherwise, Step 2 and Step 3 are repeated.

### 2.2. The NGHS Algorithm

In NGHS algorithm, three significant parameters, harmony memory considering rate (HMCR), bandwidth (BW), and pitch adjusting rate (PAR), are excluded from NGHS, and a random select rate (*p*_*m*_) is included in the NGHS. In Step 3, NGHS works as Algorithm 2, where *X* ^best^ = (*x*_1_^best^, *x*_2_^best^,…, *x*_*D*_^best^) and *X* ^worst^ = (*x*_1_^worst^, *x*_2_^worst^,…, *x*_*D*_^worst^) denote the best harmony and the worst harmony in HM, respectively. We set parameter  *p*_*m*_ = 2/*D* for the 0-1 knapsack problem and *p*_*m*_ ∈ [0.005,0.1] for continuous optimal problem.

### 2.3. The EHS Algorithm

The EHS algorithm uses the same structure with the classical HS algorithm and does not introduce any complex operations. The only difference between HS and EHS is that the EHS algorithm proposed a new scheme of tuning BW, and it works as follows (proportional to the current population variance):

(8) $$\begin{array}{c} \text{BW}=k\sqrt{\frac{1}{\text{HMS}}\sum_{i=1}^{\text{HMS}}{\left(x_{i}-\overset{-}{x}\right)}^{2}}, \end{array}$$

where *k* is the proportionality constant. If the value of *k* is high, the population variance will maintain or increase, and thus the global exploration power of algorithm will enhance. While if the value of *k* is low, the population variance will decrease and the local exploitation performance will increase. The experimental results tested by Das et al. [19] have shown that keeping *k* around 1.2 provided reasonably accurate results.

The EHS algorithm can enhance the exploratory power, and can provide a better balance between diversification and intensification. However, the exploratory power of EHS leads to the slower convergence [18], and the computational time of BW is greatly increased.

### 2.4. The ITHS Algorithm

The ITHS algorithm [18] proposed a subpopulation formation technique. The population is divided into two groups: Group A and Group B, which is carried out based on $\bar{\text{fit}}$ (the mean objective function value of all harmony vectors in HM). The harmony vectors, whose objective function value is less than $\bar{\text{fit}}$, belong to Group A, and the rest belong to Group B. The improvisation procedure of new harmony by ITHS is shown in Algorithm 3. In ITHS algorithm, the parameter PAR is updated with the number of iterations:

(9) $$\begin{array}{c} \text{PAR}=\text{PA}{\text{R}}_{\max}-\frac{\left(\text{PA}{\text{R}}_{\max}-\text{PA}{\text{R}}_{\min}\right)\times t}{T_{\max}}, \end{array}$$

where *t* and *T*_max_ are the current iterations and the maximum iterations.

## 3. HSTL Algorithm

In this section, we proposed a novel harmony search with teaching-learning strategy which derived from Teaching-Learning-Based Optimization (TLBO) algorithm. Above all, the TLBO algorithm is introduced and analyzed, and then we focus on the details of HSTL algorithm and the strategies of dynamically adjusting the parameters.

Since its origination, HS algorithm has been applied to many practical optimization problems. However, for large scale optimization problems, HS has slow convergence and low precision, which is because a new decision variable value in HM can be generated only by pitch adjustment and randomization strategies during the search process, the memory consideration rule is only used to adjust the decision variable values according to the current HM. HS can maintain a strong exploration power in the early stage, but it does not have a good exploitation in the later stage, and thus it is characterized by earlier mature and slow convergence. Therefore, for solving large scale optimization problem, the key is how to balance between global exploration performance and local exploitation ability.

### 3.1. Dimension Reduction Adjustment Strategy

As we know, for a complex optimization problem, its optimization may experience a process from extensive exploration in a large range to fine adjustment in a small range. For a *D*-dimensional optimization problem, we assume that its optimal solution is X* = (*x*_1_*, *x*_2_*,…, *x*_D_*). Let initial HM be as follows:

(10) $$\begin{array}{c} \text{HM}=\left[\begin{array}{c} X^{1}\\ X^{2}\\ \vdots \\ X^{\text{HMS}} \end{array}\right]=\left[\begin{array}{cccc} x_{1}^{1} & x_{2}^{1} & \cdots & x_{D}^{1}\\ x_{1}^{2} & x_{2}^{2} & \cdots & x_{D}^{2}\\ \vdots & \vdots & \vdots & \vdots \\ x_{1}^{\text{HMS}} & x_{2}^{\text{HMS}} & \cdots & x_{D}^{\text{HMS}} \end{array}\right]. \end{array}$$

After several iterations, the HM turns into HM2. It can be seen from HM2 that each solution *X* ^*j*^  (*j* = 1,2,…, HMS) in HM2 has nearly achieved the best solution except for one dimension (*y*_*i*_^*j*^, *j* = 1,2,…, HMS; *i* = 1,2,…, *D*):

(11) $$\begin{array}{c} \text{HM}2=\left[\begin{array}{c} X^{1}\\ X^{2}\\ \vdots \\ X^{\text{HMS}} \end{array}\right]=\left[\begin{array}{cccc} y_{1}^{1} & x_{2}^{*} & \cdots & x_{D}^{*}\\ x_{1}^{*} & y_{2}^{2} & \cdots & x_{D}^{*}\\ \vdots & \vdots & \vdots & \vdots \\ x_{1}^{*} & x_{2}^{*} & \cdots & y_{D}^{\text{HMS}} \end{array}\right]. \end{array}$$

Then we assume that only the harmony memory consideration rule of HS algorithm is employed to optimize the HM2. In the following, we employ the two methods to generate two new harmonies: *X* ^new1^ = (*x*_1_^new1^, *x*_2_^new1^,…, *x*_*D*_^new1^) and  *X* ^new2^ = (*x*_1_^new2^, *x*_2_^new2^,…, *x*_*D*_^new2^), respectively, and then analyze which method is better.

**Method 1**

Generate the solution *X* ^new1^ on each variable *x*_*i*_^new1^  (*i* = 1,2,…, *D*) by using the harmony memory consideration rule, as in Algorithm 4.

**Method 2**

Let *X* ^new2^ = *X* ^worst^ and then adjust one of the variables of the new solution  *X* ^new2^ by using the harmony memory consideration rule, as in Algorithm 5.

In the following, we analyze the two methods.

In Method 1, all of the decision variables of harmony *X* ^new1^ are chosen from HM2. If *X* ^new1^ hopes to be the optimal solution X* = (*x*_1_*, *x*_2_*,…, *x*_D_*), we should choose *x*_*i*_^new1^ = *x*_i_*, *i* = 1,2,…, *D*. In other words, *y*_*i*_^*j*^  (*j* = 1,2,…, HMS; *i* = 1,2,…, *D*) in HM2 cannot be chosen as *x*_*i*_^new1^ in the latter iteration. We can see that the probability *x*_*i*_^new1^ that turns into *x*_i_* is HMS − 1/HMS in HM2, so the probability *X* ^new1^ turns into X* = (*x*_1_*, *x*_2_*,…, *x*_D_*) is ((HMS − 1)/HMS)^*D*^.

In Method 2, assume that the *X* ^new2^ = *X* ^worst^≜(*y*_1_^1^, *x*_2_*,…, *x*_D_*), during each iteration, and only one decision variable of *X* ^new2^ needs to be adjusted with harmony memory consideration rule. So the probability that decision variable *y*_1_^1^ is chosen to adjust is 1/*D*, and then the probability that *y*_1_^1^ will be replaced with *x*_1_* is HMS − 1/HMS. Therefore, in one iteration, the probability that *X* ^new2^ turns into X* = (*x*_1_*, *x*_2_*,…, *x*_D_*) by Method 2 is 1/*D* · HMS − 1/HMS.

Next, we compare the success rate between Method 1 and Method 2 at different dimensions.

When HMS  =  10, *D* = 10, the success rate of Method 1 is

(12) P{Xnew1⟶X∗}=(HMS−1HMS)D=(10−110)10=0.34867844,

and the success rate of Method 2 is

(13) $$\begin{array}{c} P\left\{X^{\text{new}2}⟶X^{*}\right\}=\frac{1}{D}\cdot \frac{\text{HMS}-1}{\text{HMS}}=\frac{1}{10}\cdot \frac{10-1}{10}=0.09. \end{array}$$

Apparently, under this condition, Method 1 is superior to Method 2. However, if we set HMS = 10, *D* = 100, the success rate of Method 1 is

(14) P{Xnew1⟶X∗}=(HMS−1HMS)D=(100−1100)100=2.65614E−05,

while the success rate of Method 2 is

(15) P{Xnew2⟶X∗}=1D·HMS−1HMS=1100·100−1100=9.0E−03.

For different *D*, a more detailed success rate of Method 1 and Method 2 is listed in Table 1.

From Table 1, it can be seen that, for low dimensional problem (*D* < 50), success rate of Method 1 is greater than that of Method 2; however, Method 2 has higher success rate than Method 1 when *D* ≥ 50, and with the increasing of dimensionality, the success rate of Method 1 will drop dramatically, but Method 2 can maintain a higher success rate.

**Figure 1:**
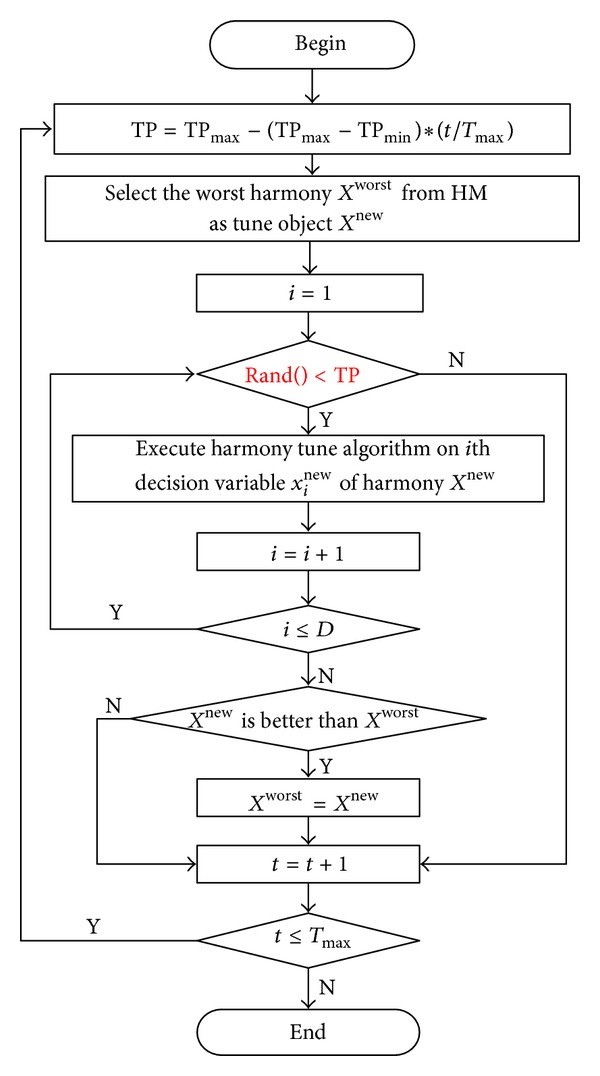
Dynamic dimension selection strategy for HS algorithm.

By above idea, in the beginning stages, exploring is on a wider domain so as to search fast, and in the later stages, the search focus on a small space to improve the accuracy of solution. Aiming at the HS algorithm, we design a dynamic dimension selection strategy to adjust some selected decision variables. A simple process is shown in Figure 1.

In Figure 1, the parameter TP represents the tune probability of each decision variable. TP changes from TP_max_ to TP_min_. In other words, each decision variable of the objective harmony vector *X* ^new^ will be tuned with probability TP which decreases from TP_min_ to TP_min_ with the increase of iterations.

### 3.2. The TLBO Algorithm

Teaching-Learning-Based Optimization (TLBO) algorithm [29–38] is a new nature-inspired algorithm; it mimics the teaching process of teacher and learning process among learners in a class. TLBO shows a better performance with less computational effort for large scale problems [31]. In addition, TLBO needs very few parameters.

In the TLBO method, the task of a teacher is to try to increase mean knowledge of all learners of the class in the subject taught by him or her depending on his or her capability. Learners make efforts to increase their knowledge by interaction among themselves. A learner is considered as a solution or a vector, different design variables of a vector will be analogous to different subjects offered to learners, and the learners' result is analogous to the “fitness” as in other population-based optimization techniques. The teacher is considered as the best solution obtained so far. The process of TLBO is divided into two phases, “Teacher Phase” and “Learner Phase.”

#### 3.2.1. Teacher Phase

Assume that there are *D* number of subjects (i.e., design variables) and *NP* number of learners (i.e., population size); *X* ^teacher^ = (*x*_1_^teacher^, *x*_2_^teacher^,…, *x*_*D*_^teacher^) is the best learner (i.e., teacher). For each learner *X* ^*j*^ = (*x*_1_^*j*^, *x*_2_^*j*^,…, *x*_*D*_^*j*^), the works of teaching are as follows:

(16) xij,new=xij,old+rand()×(xiteacher−TF×Meani),Meani=1NP∑j=1NPxij,j=1,2,…,NP,  i=1,2,…,D,

where *x*_*i*_^*j*,old^ and *x*_*i*_^*j*,new^ denote the knowledge of the *j*th learner (*X* ^*j*^) before and after learning the *i*th subject, respectively. *T*_*F*_ is the teaching factor which decides the value of mean Mean_*i*_ to be changed. *T*_*F*_ is decided by *T*_*F*_ = round[1 + rand()].

#### 3.2.2. Learner Phase

Another important approach to increase knowledge for a learner is to interact with other learners. Learning method is expressed as in Algorithm 6.

Even since the TLBO algorithm proposed by Rao et al. [29], it has been applied to the fields of engineering optimization, such as mechanical design optimization [29, 32, 37], heat exchangers [33], thermoelectric cooler [34], and unconstrained and constrained real parameter optimization problems [35, 36]. Some improved TLBO algorithm was present in last two years. An elitist TLBO algorithm for solving unconstrained optimization problems [38] by Rao and Patel and an improved harmony search based on teaching-learning strategy for unconstrained optimization problems by Tuo et al. [39] are proposed.

In the TLBO method, the teacher phase relying on the best solution found so far usually has the fast convergence speed and the well ability of exploitation; it is more suitable for improving the accuracy of the global optimal solution. Learner phase relying on other learners usually has the slow convergence speed; however, it bears stronger exploration capability for solving multimodal problems.

### 3.3. The HSTL Algorithm

In order to achieve satisfactory optimization performance by applying the HS algorithm to a given problem, we develop a novel harmony search algorithm combined teaching-learning strategy, in which both new harmony generation strategies and associated control parameter values can be dynamically changed according to the process of evolution.

It is of great importance to realize the balance between the convergence and the diversity. In the classical HS algorithm, a new harmony is generated in Step 3. After the selecting operation in Step 4, the population diversity may increase or decrease. With high population diversity, the algorithm will have strong exploration power, and at the same time the convergence and the exploitation power will decrease accordingly. Conversely, with a low population variance, the convergence and the exploitation power will increase [18]; the diversity and the exploration power will decrease. So it is significant how to keep balance between the convergence and the diversity. Classical HS algorithm loses exploitation ability easily at later evolution process [19], because of improvising new harmony from HM with a high HMCR and local adjusting with PAR. Diversity of HM decreases gradually from the early iteration to the last. Moreover, in HS algorithm, a low HMCR employed will increase the probability (1-HMCR) of random selection in search space; the exploration power will enhance, but the local search ability and the exploitation accuracy cannot be improved by single pitch adjusting strategy.

To overcome the inherent weaknesses of HS, in this section, we propose an HSTL method. In the HSTL method, an improved teaching-learning strategy is employed to improve the search ability. The HSTL algorithm works as follows.

1. Optimization target vector preparation is as follows: *X* ^new^ = *X* ^worst^, where *X* ^worst^ is the worst harmony in the current HM.
2. Improve the target vector *X* ^new^ with the following 4 strategies.

*(a) Harmony Memory Consideration.* The values of the target vector *x*_*i*_^new^  (*i* = 1,2,…, *D*) are randomly from HM with a probability of HMCR:

(17) $$\begin{array}{c} x_{i}^{\text{new}}=x_{i}^{j},\;j\in U\left\{1,2,\ldots ,\text{HMS}\right\},\;\;i=1,2,\ldots ,D. \end{array}$$

*(b) Teaching-Learning Strategy.* If the *i*th (*i* = 1,2,…, *D*) design variable of the target vector *x*_*j*_^new^ has not been considered in (a), it will learn from the best harmony (i.e., teacher) with probability TLP in the teacher phase or from the other harmony (i.e., learner) in the learner phase. The TLP is the rate of performing teaching-learning operator on design variables that have not been carried out in (a): harmony memory consideration. It works as follows.

*Teacher Phase*. In this phase, the learner will learn from the best learner (i.e., teacher) in the class. Learner modification is expressed as

(18) xinew=xinew+rand()×[xibest−TF×Mi],Mi=xiworst+xinew2, i=1,2,…,D,

where *X* ^best^ is the best harmony in HM and *X* ^worst^ is the worst harmony in HM.

The contribution of this paper is that *M*_*i*_ is replaced by (*x*_*i*_^worst^ + *x*_*i*_^new^)/2, instead of the mean value of population. This replacement will enhance diversity of population more than standard TLBO algorithm.

*Learner Phase*. Randomly select *r*_1_ and *r*_2_ from {1,2,…, HMS}, and *r*_1_ ≠ *r*_2_:

(19) If  xr1  is  better  than  xr2xinew=xinew+rand()×(xir1−xir2)Elsexinew=xinew+rand()×(xir2−xir1)Endif.

*(c) Local Pitch Adjusting Strategy*. To achieve better solutions in search space, it will carry out the local pitch adjusting strategy with probability PAR if design variables have not been selected to perform harmony memory consideration and teaching-learning strategy:

(20) $$\begin{array}{c} x_{i}^{\text{new}}=x_{i}^{\text{new}}\pm \text{rand}\left(\right)\times \text{BW}\left(i\right). \end{array}$$

*(d) Random Mutation Operator*. HSTL carries out random mutation in feasible space with probability *P*_*m*_ as follows:

(21) $$\begin{array}{c} x_{i}^{\text{new}}=x_{i}^{L}+\left(x_{i}^{U}-x_{i}^{L}\right)\times \text{rand}\left(\right). \end{array}$$

The improvisation of new target harmony in HSTL algorithm is given in Algorithm 7.

**Figure 2:**
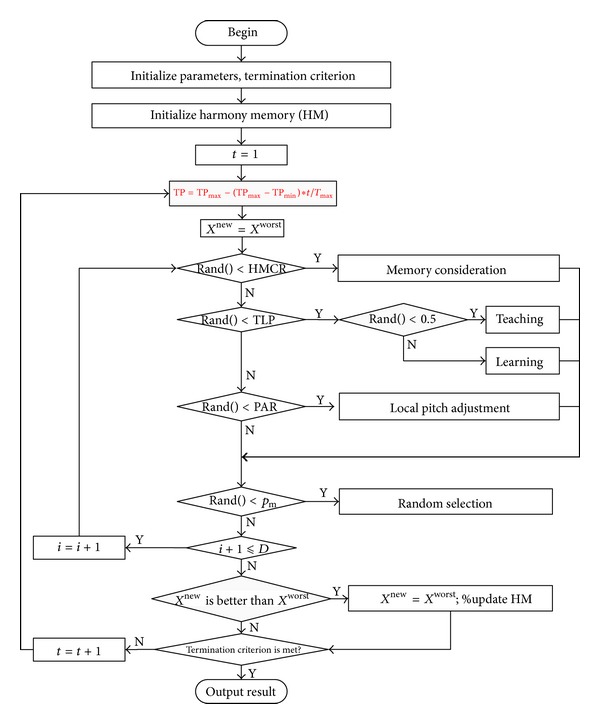
The flowchart of HSTL algorithm.

The flow chart of HSTL algorithm is shown in Figure 2. 

*(i) Update Operation.* In HSTL algorithm, update operation has some changes, as follows.

Get the best harmony *X* ^best^ and the worst harmony *X* ^worst^ from the HM:

(22) If  Xnew  is  better  than  XbestXworst=Xbest,Xbest=Xnew,ElseIf  Xnew  is  better  than  XworstXworst=Xnew,EndIf.

*(ii) Parameters Changed Dynamically.* To efficiently balance the exploration and exploitation power of the HSTL algorithm, HMCR, PAR, BW, and TLP are dynamically adapted to a suitable range with the increase of generations:

(23) HMCR=HMCRmin+(HMCRmax−HMCRmin)×(tTmax),

(24) TLP=TLPmin+(TLPmax−TLPmin)×(tTmax)k, k=5,

(25) $$\begin{array}{c} \text{PAR}=\text{PA}{\text{R}}_{\max}-\frac{\left(\text{PA}{\text{R}}_{\max}-\text{PA}{\text{R}}_{\min}\right)\times t}{T_{\max}}, \end{array}$$

(26) $$\begin{array}{c} \text{BW}=\text{B}{\text{W}}_{\max}+\exp\left[\ln\left(\frac{\text{B}{\text{W}}_{\min}}{\text{B}{\text{W}}_{\max}}\right)\times \sqrt{\frac{t}{T_{\max}}}\right], \end{array}$$

where (25) and (26) are quoted from the literature studies [18] and [16], respectively.

**Figure 3:**
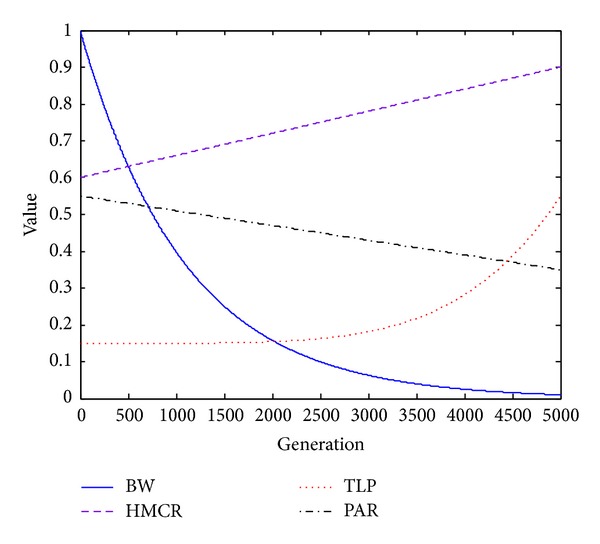
The evolution curves of parameters (HMCR, TLP, PAR, and BW) of HSTL algorithm.

Let HMCR_max_ = 0.9, HMCR_min_ = 0.6, TLP_max_ = 0.55, TLP_min_ = 0.15, PAR_max_ = 0.5, PAR_min_ = 0.33, BW_max_ = 1, and BW_min_ = 0.001. The changing curves of parameters (HMCR, TLP, PAR, and BW) are shown in Figure 3.

It can be seen that the parameter HMCR increases gradually from 0.6 to 0.9 linearly. TLP increases with low velocity in the early stage and rises sharply in the final stage. That is to say, in the beginning, the harmony consideration rule and teaching-learning strategy are carried out with a smaller probability; in the later stage, HSTL algorithm begins to focus on local exploitation with harmony consideration and teaching-learning strategy. The benefits of doing so can get more opportunities to reinforce the global exploration by strengthening disturbance in the early stage and in the final stage to intensify local search step by step, thereby acquiring high-precision solution. For the same reason, BW decreases gradually in order to reduce perturbation gradually, and the PAR's variation from 0.5 to 0.3 is to reduce the probability of pitch adjustment.

Equation (27) shows that the random mutation operation is changed dynamically from 5/D to 3/D with iterations. It can make the random disturbance change from strong to weak, and thus HSTL algorithm has strong global exploration ability in the early stage and has effective local exploitation ability in the latter stage:

(27) $$\begin{array}{c} p_{m}=\frac{5}{D}-\frac{2t/T_{\max}}{D}. \end{array}$$

## 4. Other Related Techniques for Solving 0-1 Knapsack Problem

The 0-1 knapsack problem is a large scale, multiconstraint, and nonlinear integer programming problem. So solving 0-1 knapsack problems with HSTL algorithm needs to employ constraint handling technique and integer processing method.

### 4.1. Constraint-Handling Technique

For constrained optimization problems, there have existed many successful constraint-handling techniques on solving constrained optimization problems, such as penalty function method, special representations and operators, repair method, and multiobjective method [40–53]. In this paper, because we mainly do some research on discrete optimization problems by using the HS algorithm, so special handling technique and the revision methods [23–28] for adjusting the 0-1 knapsack problems have not been adopted in this paper.

In this paper, the multiobjective method has been used for handling constrained 0-1 knapsack problems. The multiobjective method [42] is as follows.

1. Any feasible solution is better than any infeasible solution.
2. Among two feasible solutions, the one that has a better objective function value is preferred.
3. Among two infeasible solutions, the one that has a smaller degree of constraint violation is preferred. The purpose is to render the infeasible solution with large degree of constraint violation from moving gradually towards the solution with no constraint violation or with smaller degree of constraint violation.

However, for a knapsack problem with many items (*D* > 10000) and the knapsack with small capacity, if we only execute HS algorithm without any revise method to this small-capacity knapsack, all of the harmony vectors in HM are probably infeasible. In this case, even in the very good methods it is hard to obtain the feasible solutions. So, for an infeasible solution, we will do simple revise through randomly removing some item from this knapsack. In this way, the infeasible solution can gradually turn into the feasible solution.

### 4.2. Integer Processing Method

In HSTL algorithm, because the variables are adjusted by the teaching-learning strategy, local pitch adjusting strategy and random mutation operator are real numbers, so every variable is replaced by the nearest integer, that is, as follows:

(28) $$\begin{array}{c} x^{\prime }=\text{round}\left(x\right). \end{array}$$

Let *x* = (0.8,0.3,0.9,0.6,1.2,0.4). Then *x*′ = round(*x*) = (1,0, 1,1, 1,0).

## 5. Solving 0-1 Knapsack Problems with HSTL Algorithm

### 5.1. Experimental Setup and Parameters Setting

In order to evaluate the performance of the HSTL algorithm, we used a set of 13 knapsack problems (KP_1_–KP_13_). KP_1_–KP_3_ is quoted, respectively, from *f*_5_, *f*_8_ and *f*_10_ in the literature [21]. KP_4_–KP_13_ is quoted from website http://homepage.ntlworld.com/walter.barker2/Knapsack%20Problem.htm. In all the knapsack problems (KP_1_–KP_13_), KP_1_–KP_8_ are called one-dimensional problems that only include weight constraint and KP_9_–KP_13_ are called two-dimensional problems that include both weight constraint and volume constraint.

All simulation experiments are carried out to compare the optimization performance of the presented method (HSTL) with respect to (a) classical HS, (b) NGHS, (c) EHS, and (d) ITHS. In the experiments, the parameters setting for the compared HS algorithms is shown in Table 2. To make the comparison fair, the populations for all the competitor algorithms were initialized using the same random seeds. The variants of HS algorithm were set at the same termination criteria: the number of improvisations (function evaluation times: FEs) FEs = 500 × *D*, respectively. However, if the FEs > 5*E* + 05, then set FEs = 5*E* + 05.

The best and worst fitness value of each test problem are recorded for 30 independent runs; the mean fitness, standard deviation (Std), and mean runtime of each knapsack problem are calculated for 30 independent runs.

### 5.2. The Experimental Results and Analysis

**Figure 4:**
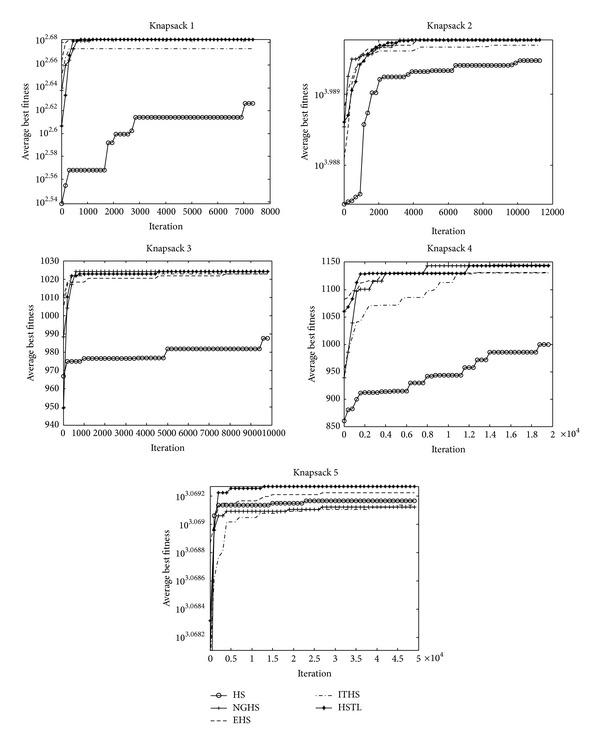
The convergence graphs of KP_1_–KP_5_.

**Figure 5:**
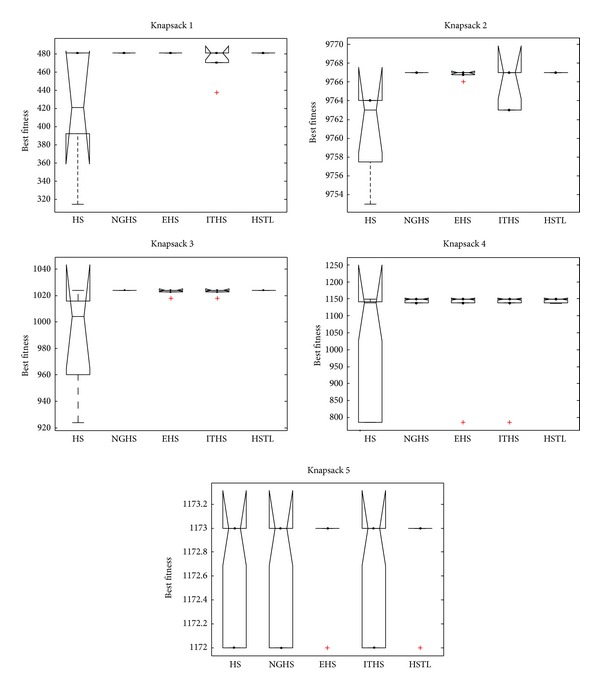
The box plots of KP_1_–KP_5_.

**Figure 6:**
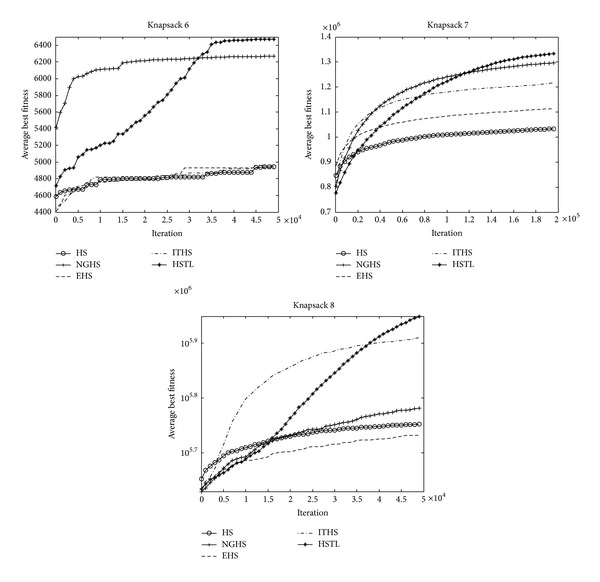
The convergence graphs of KP_6_–KP_8_.

**Figure 7:**
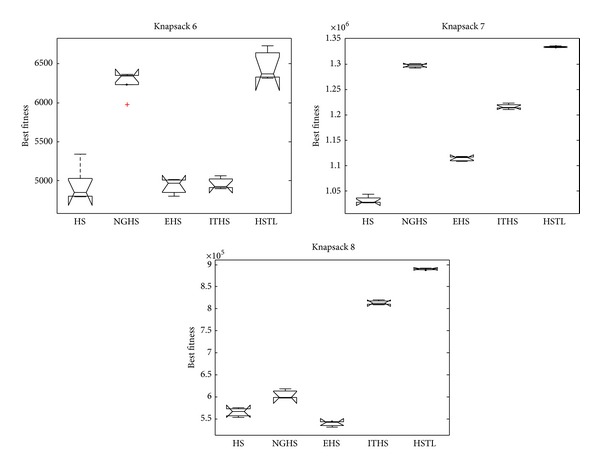
The box plots of KP_6_–KP_8_.

Table 3 reports the worst, mean, best, and Std of problem results by applying the five algorithms (HS, NGHS, EHS, ITHS, and HSTL) to optimize the knapsack problems KP_1_–KP_8_, respectively. The best results are emphasized in boldface. Figures 4 and 6 illustrate the convergence characteristics in terms of the best values of the median run of each algorithm for knapsack problems KP_1_–KP_8_. Figures 5 and 7 demonstrate the performance and stability characteristics according to the distributions of the best values of 30 runs of each algorithm for knapsack problems KP_1_–KP_8_.

Based on the resulting data in Table 3, the optimal objective values (best, mean, worst, and Std) can be easily obtained by NGHS, EHS, ITHS, and the HSTL algorithms with high accuracy on the knapsack problems KP_1_–KP_5_. However, comparatively speaking, NGHS and the HSTL algorithm have better optimal results on worst, mean, best, and Std, and thus NGHS method and the HSTL algorithm are more effective and stabilized to solve the problems KP_1_–KP_5_.

For the high-dimensional knapsack problems KP_6_–KP_8_, Table 3 shows that the HSTL algorithm has obvious advantages over other variants of HS algorithms. Comparing with other HS algorithms, although HSTL algorithm has slow convergence speed in the early stage, it can be constant to optimize the solutions and obtain high precision solutions in the latter stage, which can be seen from Figure 6.

It is evident from Figure 7 that HSTL algorithm has better convergence, stability, and robustness in most cases than HS, NGHS, EHS, and ITHS algorithms.

**Figure 8:**
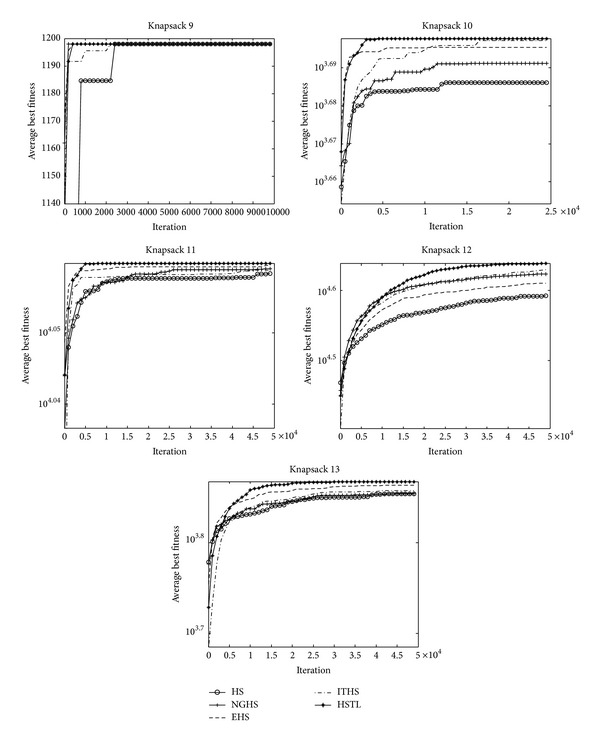
The convergence graphs of KP_9_–KP_13_.

**Figure 9:**
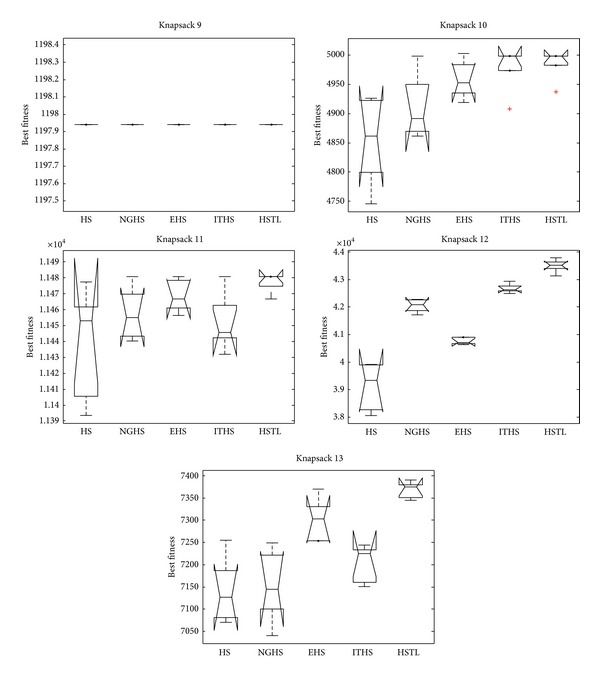
The box plots of KP_9_–KP_13_.

Table 4 shows the experimental results on algorithms HS, NGHS, EHS, ITHS, and HSTL for two-dimensional knapsack problems: KP_9_–KP_13_.  Figure 8 shows the convergence graphs, and Figure 9 is the box plots of independent 30 runs of knapsack problems KP_9_–KP_13_.

It can be seen evidently from Table 4 that the HSTL algorithm attained the optimal best, mean, worst, and Std results among all two-dimensional knapsack problems.

From the convergence graphs (Figure 8), it can be seen that the HSTL algorithm has a strong search ability and convergence throughout the search process for the two-dimensional knapsack problems. As can be seen from the box plots (Figure 9), the HSTL has demonstrated some advantage over the other four algorithms on solving two-dimensional 0-1 knapsack problems.

## 6. Conclusion

In this paper, a novel harmony search with teaching-learning strategies is presented to solve 0-1 knapsack problems. The HSTL algorithm employs the idea of teaching and learning. Four strategies are used to maintain the proper balance between exploration power and exploitation power. With the process of evolution, the dynamic strategy is adopted to change the parameters HMCR, TLP, BW, and PAR. Experimental results showed that the HSTL algorithm has stronger ability to resolve the high-dimensional 0-1 knapsack problems. However, the HSTL algorithm has more parameters. In the future, we should focus on improving the structure of HSTL algorithm, decreasing the running cost and enhancing efficiency for solving complex optimization problems.

## Figures and Tables

**Algorithm 1:**
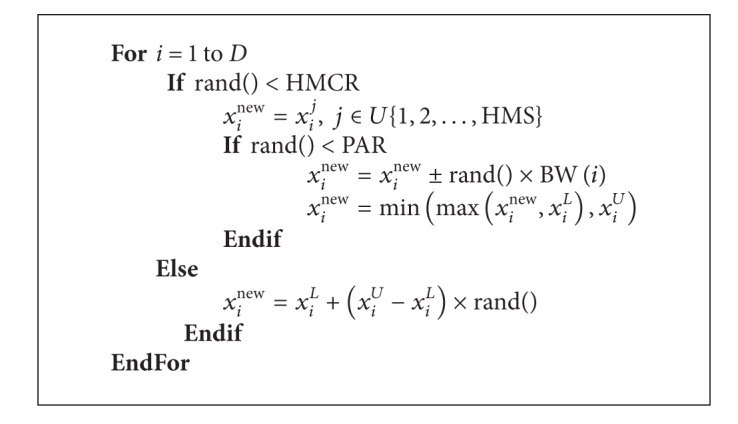
The improvisation procedure of new harmony by classical HS.

**Algorithm 2:**
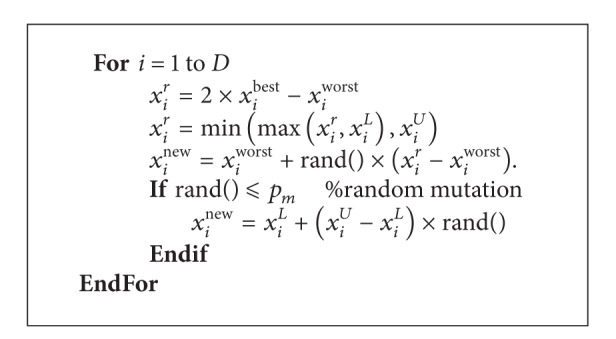
The improvisation procedure of new harmony by NGHS.

**Algorithm 3:**
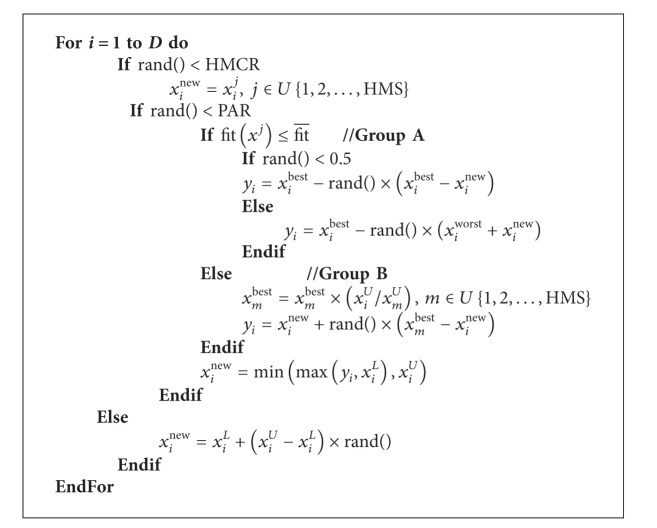
The improvisation procedure of new harmony by ITHS.

**Algorithm 4:**
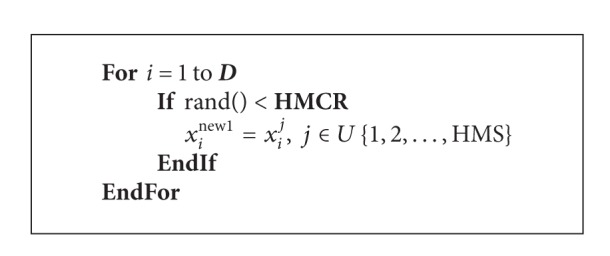


**Algorithm 5:**
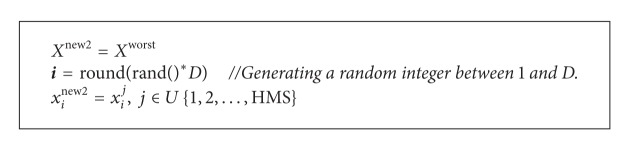


**Algorithm 6:**
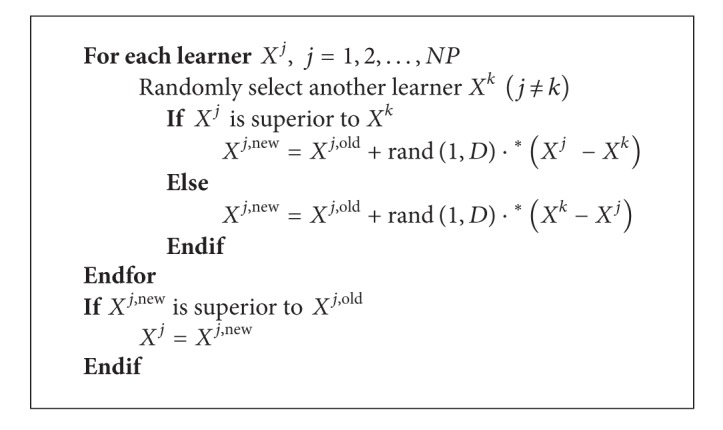
The procedure of learner phase.

**Algorithm 7:**
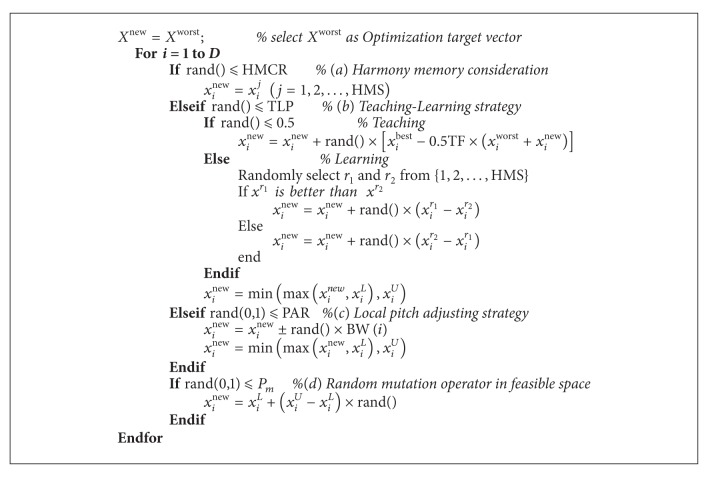
Improvisation of new harmony in HSTL algorithm.

**Table 1:** Success rate of Methods 1 and 2.

HMS	*D *	The success rate	
		Method 1	Method 2
10	10	3.48678*E* − 01	9.00000*E* − 02
10	20	1.21577*E* − 01	4.50000*E* − 02
10	30	4.23912*E* − 02	3.00000*E* − 02
10	40	1.47809*E* − 02	2.25000*E* − 02
10	50	5.15378*E* − 03	1.80000*E* − 02
10	60	1.79701*E* − 03	1.50000*E* − 02
10	70	6.26579*E* − 04	1.28571*E* − 02
10	80	2.18475*E* − 04	1.12500*E* − 02
10	90	7.61773*E* − 05	1.00000*E* − 02
10	100	2.65614*E* − 05	9.00000*E* − 03
10	110	9.26139*E* − 06	8.18182*E* − 03
10	120	3.22925*E* − 06	7.50000*E* − 03
10	130	1.12597*E* − 06	6.92308*E* − 03
10	140	3.92601*E* − 07	6.42857*E* − 03
10	150	1.36891*E* − 07	6.00000*E* − 03
10	160	4.77311*E* − 08	5.62500*E* − 03
10	170	1.66428*E* − 08	5.29412*E* − 03
10	180	5.80299*E* − 09	5.00000*E* − 03
10	190	2.02338*E* − 09	4.73684*E* − 03
10	200	7.05508*E* − 10	4.50000*E* − 03
10	300	1.87393*E* − 14	3.00000*E* − 03
10	400	4.97741*E* − 19	2.25000*E* − 03
10	500	1.32207*E* − 23	1.80000*E* − 03
10	600	3.51161*E* − 28	1.50000*E* − 03
10	700	9.32731*E* − 33	1.28571*E* − 03
10	800	2.47747*E* − 37	1.12500*E* − 03
10	900	6.58049*E* − 42	1.00000*E* − 03
10	1000	1.74787*E* − 46	9.00000*E* − 04

**Table 2:** Parameter settings for the compared HS algorithms (HS, NGHS, EHS, ITHS, and HSTL).

Algorithm	HMS	HMCR	PAR	BW	others
HS	5	0.99	0.33	BW = (*X* ^*U*^ − *X* ^*L*^)/1000	/
NGHS	5	/	/	/	/
EHS	50	0.99	0.33	$\text{BW}=1.17\sqrt{\left(1/\text{HMS}\right)\sum_{i=1}^{\text{HMS}}{\left(x_{i}-\overset{-}{x}\right)}^{2}}$	/
ITHS	10	0.99	0.33	BW_max_ = (*X* ^*U*^ − *X* ^*L*^)/2 BW_min_ = (*X* ^*U*^ − *X* ^*L*^)/10	/
HSTL	10	HMCR_max_ = 0.95 HMCR_min_ = 0.6	PAR_max_ = 0.5 PAR_min_ = 0.2	BW_max_ = (*X* ^*U*^ − *X* ^*L*^)/2 BW_min_ = (*X* ^*U*^ − *X* ^*L*^)/10	TLP_max_ = 0.55, TLP_min_ = 0.15

**Table 3:** The result of 1-dimensional (weight versus value) knapsack problems.

Problem	*D *	Target weight	Total weight	Total values	Optimal result	Algorithm	Outcomes				Runtime
							Worst	Mean	Best	Std	
KP_1_	15	375	741.9172	62.9963	**481.069**	HS	314.9297	423.191	**481.069**	67.92161	0.169532
						NGHS	**481.069**	**481.069**	**481.069**	6.4***E ***−14	0.173909
						EHS	**481.069**	**481.069**	**481.069**	6.4***E ***−14	0.814028
						ITHS	437.9345	472.4424	**481.069**	19.2905	0.447476
						HSTL	**481.069**	**481.069**	**481.069**	6.4***E ***−14	0.330757

KP_2_	23	10000	19428	19309	**9767**	HS	9747	9760	**9767**	5.533596	0.313597
						NGHS	**9767**	**9767**	**9767**	**0**	0.305331
						EHS	9643	9751.6	**9767**	22.64509	1.595463
						ITHS	9756	9765.7	**9767**	2.641186	0.790986
						HSTL	**9767**	**9767**	**9767**	**0**	0.585312

KP_3_	20	878	1098	1085	**1024**	HS	924	987.4	**1024**	40.395544	0.254834
						NGHS	**1024**	**1024**	**1024**	**0**	0.245683
						EHS	1018	1022.8	**1024**	2.6832816	1.274226
						ITHS	1018	1022.8	**1024**	2.6832816	0.654352
						HSTL	**1024**	**1024**	**1024**	**0**	0.485799

KP_4_	40	15	374	14049	**1149**	HS	786	1109.4	**1149**	113.7494	12.51123
						NGHS	**1138**	**1147.9**	**1149**	**3.478505**	12.72128
						EHS	786	1109.4	**1149**	113.7494	15.64561
						ITHS	786	1112.7	**1149**	114.7907	13.6885
						HSTL	**1138**	**1147.9**	**1149**	**3.478505**	13.29233

KP_5_	100	27	1360	34965	**1173**	HS	1172	1172.5	**1173**	0.508548	37.14283
						NGHS	1172	1172.633	**1173**	0.490133	37.22416
						EHS	1172	1172.567	**1173**	0.504007	53.20237
						ITHS	1172	1172.567	**1173**	0.504007	41.50243
						HSTL	1172	**1172.9**	**1173**	**0.305129**	37.11053

KP_6_	10000	431	349354	3011792	**unknown**	HS	4795	4941	5338	228.20495	2902.844
						NGHS	5976	6270.4	6363	165.43821	1881.969
						EHS	4797	4929.8	5014	94.099416	16979.43
						ITHS	4899	4960.6	5064	72.081898	5047.957
						HSTL	**6318**	**6474.4**	**6730**	**185.7156**	**1506.648**

KP_7_	10000	1765326	5033006	2063406	**unknown**	HS	1068628	1070926.5	1073225	3250.5699	4526.0894
						NGHS	1126584	1127050.5	1127517	659.73063	5060.0062
						EHS	1041435	1043733.5	1046032	3250.5699	5435.1693
						ITHS	1124261	1129389	1134517	7252.0871	4555.54
						HSTL	**1193743**	**1194524**	**1195304**	**1103.794**	**3326.1473**

KP_8_	11000	1000000	5526981	2263955	**unknown**	HS	529153	532801.8	537353	3001.1646	10633.825
						NGHS	738654	762855.4	798991	26865.404	11912.341
						EHS	480038	486268.8	490627	4481.0616	13084.292
						ITHS	795202	800908	807164	4864.5424	10785.623
						HSTL	**940656**	**944780**	**947850**	**2630.108**	**9606.735**

**Table 4:** The result of 2-dimensional (weight and volume versus value) knapsack problems.

Problem	*D *	Target weight ∣ volume	Total weight	Total volume	Total values	Optimal result	Algorithm	Outcomes				Runtime
								Worst	Mean	Best	Std	
KP_9_	20	20 | 25	111.63	170.27	3813.6	**1197.94**	HS	**1197.94**	**1197.94**	**1197.94**	0	**7.90831**
							NGHS	**1197.94**	**1197.94**	**1197.94**	0	8.092175
							EHS	**1197.94**	**1197.94**	**1197.94**	0	8.978277
							ITHS	**1197.94**	**1197.94**	**1197.94**	0	8.588568
							HSTL	**1197.94**	**1197.94**	**1197.94**	0	8.31578

KP_10_	50	114 | 133	400.56	408.57	10211	**5002.81**	HS	4849.19	4935.062	**5002.81**	45.16856	19.03619
							NGHS	4917.42	4963.421	**5002.81**	32.60289	19.15758
							EHS	4933.24	4983.169	4998.27	24.18182	23.48809
							ITHS	4926.07	4975.32	**5002.81**	29.72861	20.82369
							HSTL	**4995.48**	**5000.063**	**5002.81**	**2.981432**	**18.9528**

KP_11_	100	1500 | 3000	3090.5	4599.2	15278	**11480.84**	HS	11393.59	11438.024	11477.52	35.010842	51.07192
							NGHS	11440.36	11457.378	**11480.84**	16.483244	51.2287
							EHS	11456.44	11468.79	**11480.84**	10.221502	65.89753
							ITHS	11431.96	11452.068	**11480.84**	18.277327	54.87239
							HSTL	**11466.63**	**11477.33**	**11480.84**	**6.153989**	**45.31429**

KP_12_	1000	30000 | 40000	250320	400090	157890	**unknown**	HS	38064.94	39108.266	39924.89	866.36169	414.50957
							NGHS	41709.51	42048.386	42280.62	235.6772	424.71994
							EHS	40627.36	40766.678	40903.3	123.45544	454.46526
							ITHS	42485.83	42671.15	42928.94	163.78948	439.51866
							HSTL	**43953.97**	**44119.987**	**44409.66**	**119.80076**	**393.5962**

KP_13_	300	800 | 700	13605	14157	48464	**unknown**	HS	7070.4	7140.222	7254.76	73.854458	36.347165
							NGHS	7040.92	7153.61	7249.1	81.266043	36.41226
							EHS	7253.67	7299.32	7369.58	48.490527	39.440654
							ITHS	7150.24	7202.6	7244.01	42.540662	39.450648
							HSTL	**7345.46**	**7367.95**	**7390.52**	**18.42674**	**29.889155**

Bold indicates best results.
